# Somatotopic Arrangement of the Human Primary Somatosensory Cortex Derived From Functional Magnetic Resonance Imaging

**DOI:** 10.3389/fnins.2020.598482

**Published:** 2021-01-07

**Authors:** W. R. Willoughby, Kristina Thoenes, Mark Bolding

**Affiliations:** ^1^Department of Radiology, The University of Alabama at Birmingham, Birmingham, AL, United States; ^2^Department of Neurobiology, The University of Alabama at Birmingham, Birmingham, AL, United States

**Keywords:** homunculus, fMRI, somatosensory, topographic maps, MR safe

## Abstract

Functional magnetic resonance imaging (fMRI) was used to estimate neuronal activity in the primary somatosensory cortex of six participants undergoing cutaneous tactile stimulation on skin areas spread across the entire body. Differences between the accepted somatotopic maps derived from Penfield's work and those generated by this fMRI study were sought, including representational transpositions or replications across the cortex. MR-safe pneumatic devices mimicking the action of a Wartenberg wheel supplied touch stimuli in eight areas. Seven were on the left side of the body: foot, lower, and upper leg, trunk beneath ribcage, anterior forearm, middle fingertip, and neck above the collarbone. The eighth area was the glabella. Activation magnitude was estimated as the maximum cross-correlation coefficient at a certain phase shift between ideal time series and measured blood oxygen level dependent (BOLD) time courses on the cortical surface. Maximally correlated clusters associated with each cutaneous area were calculated, and cortical magnification factors were estimated. Activity correlated to lower limb stimulation was observed in the paracentral lobule and superomedial postcentral region. Correlations to upper extremity stimulation were observed in the postcentral area adjacent to the motor hand knob. Activity correlated to trunk, face and neck stimulation was localized in the superomedial one-third of the postcentral region, which differed from Penfield's cortical homunculus.

## 1. Introduction

Large portions of the mammalian cerebral cortex are devoted to processing sensory inputs, offering investigators a solid starting point for understanding functional organization in the brain (Krubitzer and Seelke, 2012). Initially, neuroscientists were forced to use highly invasive methods to probe the mechanisms of sensation, which limited the applicability of such studies to humans (Penfield and Boldrey, 1937; Hubel and Wiesel, 1959; Brown-Sequard, 1968; Robinson et al., 1978; Manger et al., 1997). Advances in functional neuroimaging over the past several decades have opened new avenues for investigating sensation and perception non-invasively, making these techniques suitable for routine studies in healthy humans (Lawrence et al., 2008; Sanchez-Panchuelo et al., 2010, 2014; Stringer et al., 2011; Kolasinski et al., 2016; Akselrod et al., 2017; Schluppeck et al., 2018; Da Rocha Amaral et al., 2019; Kaas et al., 2019; Kessner et al., 2019; Mancini et al., 2019; Van Essen et al., 2019; Allen et al., 2020; Luijten et al., 2020; Puckett et al., 2020).

A large body of work has been published that pertains to the evolution, structure, and function of the somatosensory nervous systems of mammals (Dreyer et al., 1975; Beck et al., 1996; Manger et al., 1997; Krubitzer and Seelke, 2012; Van Essen et al., 2019), including humans (Penfield and Boldrey, 1937; Brown-Sequard, 1968; Woolsey et al., 1979; Baumgartner et al., 1991; Engel et al., 1997; Arichi et al., 2010; Dall'Orso et al., 2018). Much of what is known about functional localization in the somatosensory cortex has been learned through lesion studies, electrical recording of neuronal activity through implanted electrodes (Dreyer et al., 1975; Beck et al., 1996; Manger et al., 1997; Lipton et al., 2010), and direct electrical stimulation of exposed brain tissue of conscious subjects (Penfield and Boldrey, 1937; Woolsey et al., 1979; Roux et al., 2018). In the time since Wilder Penfield and his colleagues published the results of their intraoperative experiments in the 1930s, the cartoon of the cortical homunculus has become pervasive in the field of neuroscience, despite the seminal neuroscientists' early warnings about reproducibility (Penfield and Boldrey, 1937; Snyder and Whitaker, 2013). Follow-up studies conducted since then have mostly supported the early findings of a somatotopic sequence in the Rolandic cortex (Roux et al., 2018), but there have been relatively few studies measuring individual, inter-subject differences in the functional anatomy of the somatosensory system (Sanchez-Panchuelo et al., 2010, 2014; Kolasinski et al., 2016). Perhaps more importantly, the homunculus was revealed from experiments in which the input-output pathways were dramatically different from normal physiology, bypassing the entire peripheral nervous system. The implications of these differences are not completely clear and cannot be explored until a method of routine, non-invasive measurement of brain function resulting from peripheral tactile stimulation is developed.

Direct microelectrode recording and other invasive methods are not practical for routine study of the living human cortex. Human *in vivo* studies have been restricted by either a small pool of subjects eligible for intracranial operative measurements or by the technological limitations of less invasive neuroimaging techniques. Functional magnetic resonance imaging (fMRI) is sensitive to changes in nuclear spin relaxation times due to paramagnetic effects of oxygenated hemoglobin on nearby protons. However, fMRI measures the effects of neurovascular coupling, which is indirectly related to neuronal activity. fMRI has good spatial resolution of about 1–2 mm, and advances in multiband pulse sequences have improved the temporal resolution of this technique to <2 s. The spatial and temporal resolution makes fMRI our neuroimaging technique of choice for mapping individual differences in sensory somatotopic maps of human subjects.

We hypothesized that somatosensory mapping experiments of spatially distant skin areas using fMRI may generate cortical maps that differ from those produced by experiments that applied electrical stimulation directly to cortical tissue. Specifically, transposed representations or evidence of multiple representation of a single body part on the cortex were sought. Several fMRI studies have been published that have mapped somatosensory cortex of humans and that tend to agree with the prevailing homunculus model (Servos et al., 1999; Backes et al., 2000; Sanchez-Panchuelo et al., 2010; Besle et al., 2013; Wardman et al., 2014; Schweisfurth et al., 2018; Da Rocha Amaral et al., 2019; Luijten et al., 2020; O'Neill et al., 2020). Few fMRI studies seem to have addressed whole body sensory somatotopy, so this work helps to fill that knowledge gap (Sanchez-Panchuelo et al., 2018; Saadon-Grosman et al., 2020). To perform the experiments, a pneumatic stimulation device was built that is safe to use in an MR environment. The device simulates the action of a Wartenberg pinwheel (Wartenberg, 1937) over a small area of skin, but is made of plastic and does not puncture the skin or elicit pain. The device was fully automated and was synchronized to the acquisition of fMRI data, which enabled the use of analytical techniques based on stimulus timing and temporal correlation that have been used to study topographic maps of the visual and somatosensory systems (Freeman et al., 2011; Sanchez-Panchuelo et al., 2018).

## 2. Methods

### 2.1. Ethics and Participants

The protocol for this study was approved by UAB's institutional ethics review board (IRB). Seven healthy participants (3 female, 4 male, ages 19–34) with no MRI contraindications or previous history of neurological disorders were recruited and gave their voluntary, informed consent to participate.

### 2.2. Pneumatic Light Touch Stimulation

**Figure 1:**
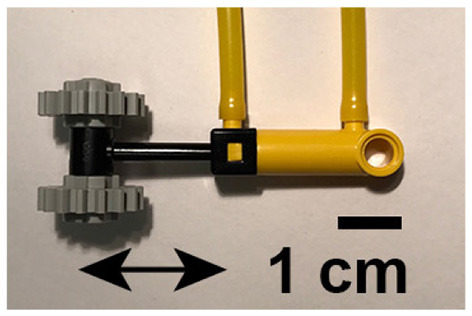
Photograph of one of the eight pneumatic tactile stimulators used for somatosensory topographic mapping experiments. Arrows depict direction of travel of toothed wheels. Black line shows 1 cm scale.

A pneumatic device was developed and built to automate and synchronize tactile stimulation. Light touch stimuli were produced by alternatingly pressurizing and depressurizing opposite ends of 8 plastic pneumatic cylinders (LEGO part x189c01) and driving plastic pistons back and forth. Two small toothed wheels were attached to each of the eight pistons. A photograph of one of the devices is shown in Figure 1. The stimulators were capable of delivering light touch to a 4 cm^2^ area of skin. Each cylinder was driven with a 5 port, 2-way valve connected using lengths of polyurethane tubing. The state of each valve was switched independently with a 24 V solenoid using a microcontroller (Arduino MEGA) and 5 V relays. Constant air pressure of 200 kPa was provided to each valve by an air compressor.

The beginning of a stimulation sequence was triggered by a TTL pulse output from the MRI scanner at the start of fMRI volume acquisition, ensuring synchronicity between stimulus presentation and acquisition of functional data. Each stimulator was attached to the body using elastic bands, and self-adhesive bandages were used in more sensitive areas to prevent discomfort from pinching. Before each experiment and after being placed inside the bore of the scanner, the participant was asked to verbally announce which skin area was being stimulated as a stimulation sequence was run. This was done to ensure that each stimulator was attached as intended and received adequate air pressure to induce sensation.

### 2.3. Paradigm

**Figure 2:**
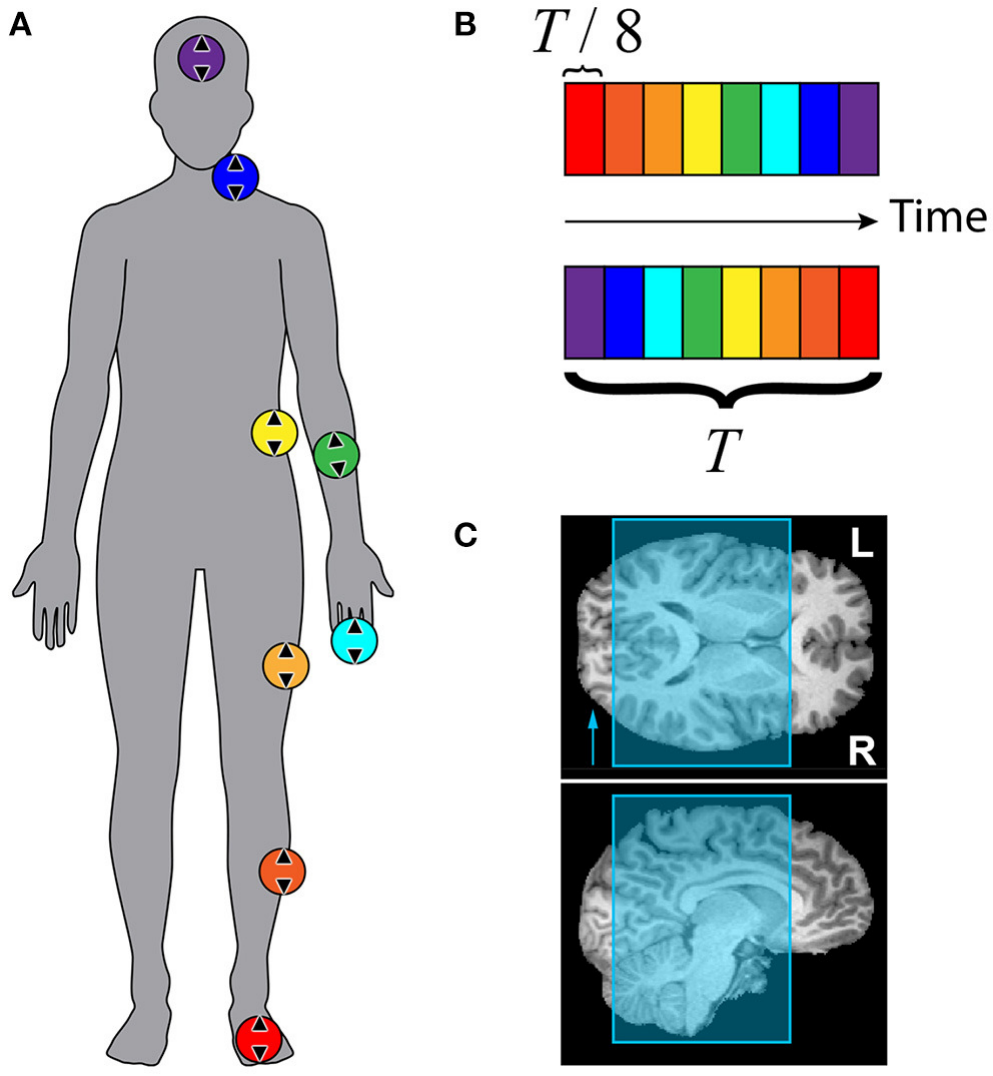
Stimulation paradigm and scan coverage for somatosensory mapping with fMRI. **(A)** Colored circles represent approximate stimulator locations: glabella, neck near collarbone, anterior forearm, middle fingertip, trunk beneath ribcage, thigh, shin, and volar side of foot. Arrows within circles show approximate orientation of linear actuators. **(B)** Colored boxes depict timing of tactile stimuli. Top: forward sequence. Bottom: reverse sequence. Total stimulation period *T* was 72 s (40 s for subject F). Each stimulator was active for *T*/8 = 9 s (5 s for subject F) before switching to the next. Each run consisted of 5 stimulation periods (9 for subject F). **(C)** EPI slice coverage overlaid on T1-weighted MR images. Slices were stacked along the anterior-posterior axis. Phase encoding direction was right-left, indicated by the arrow in the axial view.

Figure 2 summarizes the traveling wave paradigm that was used to carry out the somatosensory mapping for each subject. Eight stimulators were attached to eight disparate skin areas on the participant's body, all but one of which were placed with left laterality, as shown in Figure 2A. Colored circles depict the locations of each of the stimulators: (1) the middle of the inferior side of the left foot, (2) the lateral side of the left shin, approximately halfway between the ankle and knee, (3) the lateral side of the left thigh, approximately halfway between the knee and hip, (4) the left lateral side of the torso at approximately the bottom of the rib cage, (5) the anterior side of the left forearm, approximately halfway between the elbow and wrist, (6) the distal phalanx of the left middle finger, (7) the left lateral side of the neck, at the approximate level of the C4 vertebra, and (8) the middle of the forehead, on the glabella. The MRI receive coil and headphones did not allow for comfortable placement of the last stimulator on the left side of the forehead. Foot, forearm, finger, neck, and forehead stimulators were in direct contact with the skin, while the shin, thigh, and trunk stimulators were placed over participants' scrubs. The direction of linear movement for each stimulator is shown using arrows inside each of the colored circles.

Figure 2B illustrates stimulator timing for forward and reverse stimulation sequences. Each colored block represents the time interval over which the stimulator with matching color is active. Only one stimulator was active at any given time. The time axis runs horizontally from left to right. The top sequence was defined as the “forward” sequence, and the bottom sequence was defined as the “reverse” sequence. The first stimulator began its cycle at the start of data collection at the beginning of the fourth repetition time (TR). Each functional imaging run lasted for 5 stimulation cycles that were each 72 s, for a total of 6 min per scan. Four scans were acquired for each participant, two using the forward sequence (foot to head) and two using the reverse sequence (head to foot). For subject F, the stimulation cycle was 40 s, and 9 cycles were completed for each 6 min scan.

### 2.4. MRI Acquisition

MR imaging was carried out at 3T using a Prisma scanner (Siemens Healthineers, Erlangen, Germany). A T1-weighted, magnetization-prepared rapid gradient echo (MPRAGE) 3D imaging sequence with 1 mm isotropic voxels was used to acquire high-resolution images for co-registration of echo-planar imaging (EPI) volumes and for cortical parcellation and surface reconstructions. Functional blood oxygen level dependent (BOLD) imaging was done using a T2*-sensitive, multi-band, single-shot 2D gradient echo EPI sequence (CMRR, University of Minnesota). The TR was 2 s, during which 50 contiguous 2 mm coronal slices were acquired. The echo time (TE) was 35 ms, and the flip angle was 75°. In-plane resolution was set by a 96 × 96 acquisition matrix and a 192 mm field of view (FOV), yielding 2 mm isotropic voxels. Phase encoding was in the right to left direction, and the multi-band acceleration factor was 2 with no in-plane acceleration. Data acquisition began after three TRs were discarded by the scanner to ensure the MR signal had reached steady state. Five functional volumes with phase encoding in the right to left and left to right directions were acquired at the end of each scanning session for EPI distortion correction.

### 2.5. Data Processing

Functional EPI data was processed using AFNI (NIMH) and FreeSurfer (http://surfer.nmr.mgh.harvard.edu/) (Cox, 1996; Dale et al., 1999; Saad et al., 2001). Cortical parcellation and generation of surface meshes with convexity maps were done using FreeSurfer. Slice timing alignment used AFNI's 3dTshift function, and distortion correction was applied using the blip up-down technique built into the afni_proc.py script. Functional volumes were spatially registered to the volume with the least amount of motion for that run, each of which was in turn registered to the high-resolution anatomical image using a signed local Pearson correlation cost functional. fMRI volumes were censored if motion was estimated to be ≥0.3 mm. If more than 25% of volumes were censored, the entire dataset was discarded. Due to these exclusion criteria, one participant was not included in the analysis. Data from the remaining six participants were analyzed.

BOLD time courses were mapped onto each subject's cortical surface using AFNI's 3dVol2Surf function. Values from voxels lying on a line connecting adjacent nodes of the outer white matter surface mesh and the pial surface mesh were averaged to produce a time course for that particular surface element. A region of interest (ROI) encompassing the central sulcus, postcentral gyrus, postcentral sulcus, and paracentral lobule was defined on the surface using the parcellation atlas generated by FreeSurfer. Co-registration of individual cortical surfaces to the surface of the Colin 27 template brain was done using standardized meshes (Holmes et al., 1998; Saad and Reynolds, 2012). This process allowed direct comparison of individual results in a common MNI coordinate system. After surface registration, data were spatially filtered using a Gaussian kernel with a 4 mm full-width at half-maximum. The time courses were then detrended by subtracting third-order polynomials fit to each time course by least squares regression. Finally, average time courses were calculated from the two functional runs that used the forward stimulation sequence and from the two runs that used the reverse sequence.

**Figure 3:**
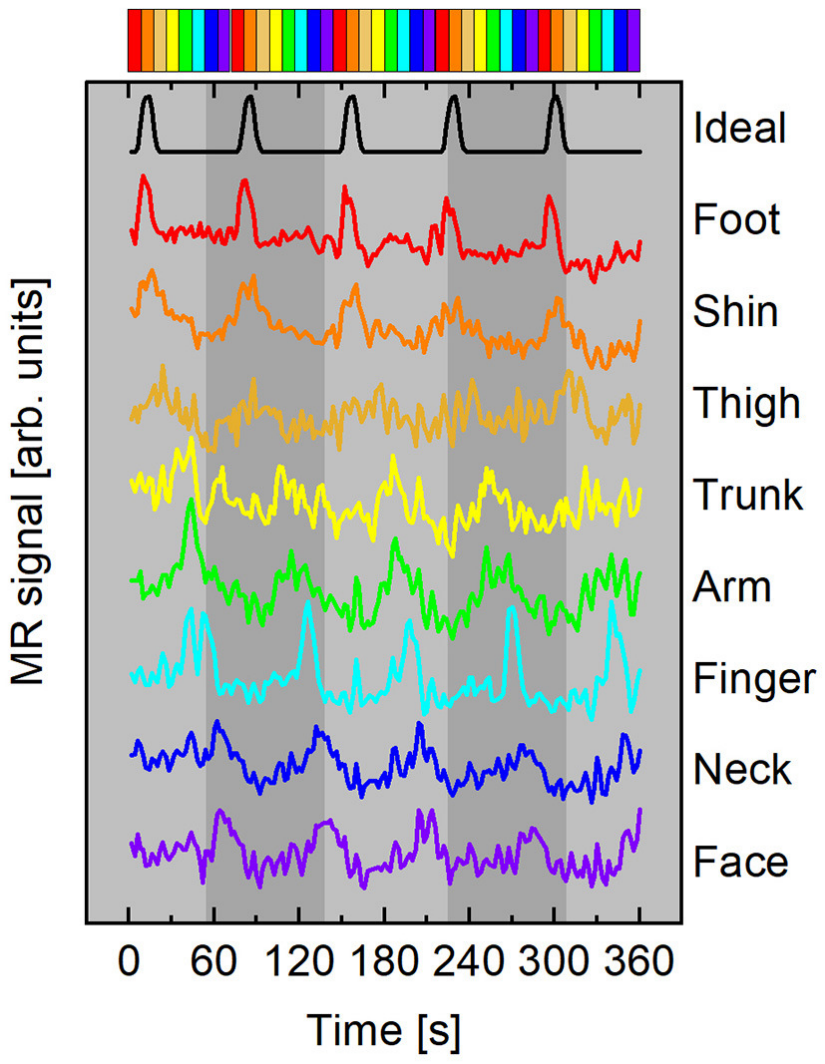
Representative mean time courses (subject A) averaged from surface elements in ROI with ρ ≥ 0.45. Curves color-coded according to time delay, Δ*t*, with respect to the ideal time course (black curve). Time courses have been vertically offset for clarity. Top: Colorbar summarizing stimulus timing for the forward paradigm depicted in Figure 2.

AFNI's 3d delay function was used to estimate temporal cross-correlation coefficients between the measured response of a surface element *r*(*t*) and an ideal response *s*(*t*) based on stimulus timing and a canonical hemodynamic response function (HRF). A gamma variate function

(1) $$\begin{array}{l} f\left(t\right)={\left(\frac{t}{pq}\right)}^{p}{\text{e}}^{p-t/q} \end{array}$$

was used for the HRF, where *p* = 8.6, *q* = 0.547. *f*(*t*) was convolved with a boxcar function *b*(*t*) with duration 9 s (5 s for subject F), beginning at *t* = *t*_0_ and repeating every stimulation period, *T* = 72 s (40 s for subject F). To avoid reporting delays >10% of each time series' total length of 360 s, an ideal time course was generated for each stimulus. The initial time of stimulation for each *s*_*i*_(*t*) was *t*_0_ = *T*(*i* − 1)/8 s, where *i*∈{1, 2, 3, 4, 5, 6, 7, 8} represents which stimulator was active in the sequence. This ensured that increases in delay variance due to the spectral characteristics of fMRI noise were mitigated (Saad et al., 2001). The ideal time course *s*_1_(*t*) beginning at *t* = 0 is plotted at the top of Figure 3.

Cross-correlation coefficients and phase delays were estimated for all of the surface element time courses for each of the four runs using eight ideal courses *s*_*i*_(*t*), yielding 32 datasets for each participant. Any time delays greater than the length of one stimulation block (9 s for subjects A–E, 5 s for subject F) were masked out by setting ρ = 0 for that stimulus-response pair. For example, if a time course from a forward stimulation sequence (run 1) was found to be maximally correlated to the ideal time course *s*_1_(*t*) with a time delay of 1 s, then the surface element for that dataset (run 1-ideal 1) would be mapped to the foot. If, however, the time delay was >9 s, then that surface element would be masked out by setting ρ to zero. The correlation-delay datasets estimated from forward and reverse stimulation sequences were combined in a “winner takes all” manner, such that if a cortical surface element correlated to more than one time delay, it was assigned the delay with the greatest correlation. Next, time delays estimated by the 3ddelay algorithm for each of the 16 combined datasets had to be shifted by *t*_0_ = *T*(*i* − 1)/8 s in order to reflect the total delay from *t* = 0. Finally, the correlation coefficients and corrected delays estimated from each *s*_*i*_(*t*) were combined in the same “winner takes all” approach to generate a complete map. If a surface element was correlated to more than one ideal time course at this stage, then the delay estimated from the ideal time course *s*_*i*_ with the greatest correlation coefficient was assigned to that surface element. Time delays are reported in terms of a phase shift Δϕ from the ideal time series *s*_1_(*t*) in radians, where Δϕ = (2π/*T*)Δ*t*.

### 2.6. Statistical Analysis

Surface clusters were calculated using AFNI's SurfClust function. The maximum number of surface elements between a correlated surface element and the cluster to which it was assigned was 10. The correlation threshold for surface elements to be considered significantly correlated to stimuli was determined from the histogram of cross-correlation coefficients for every surface element, including those outside the ROI. A significance level of 0.05 was used for the minimum cross-correlation coefficient of an activated surface element, denoted ρ_*threshold*_. The center of mass of each cluster with area >2 mm^2^ was calculated by minimizing the weighted sum of vectors joining each surface element to the center of mass surface element. Vectors of each surface element were weighted by their correlation coefficient.

## 3. Results

### 3.1. Time Courses

**Figure 4:**
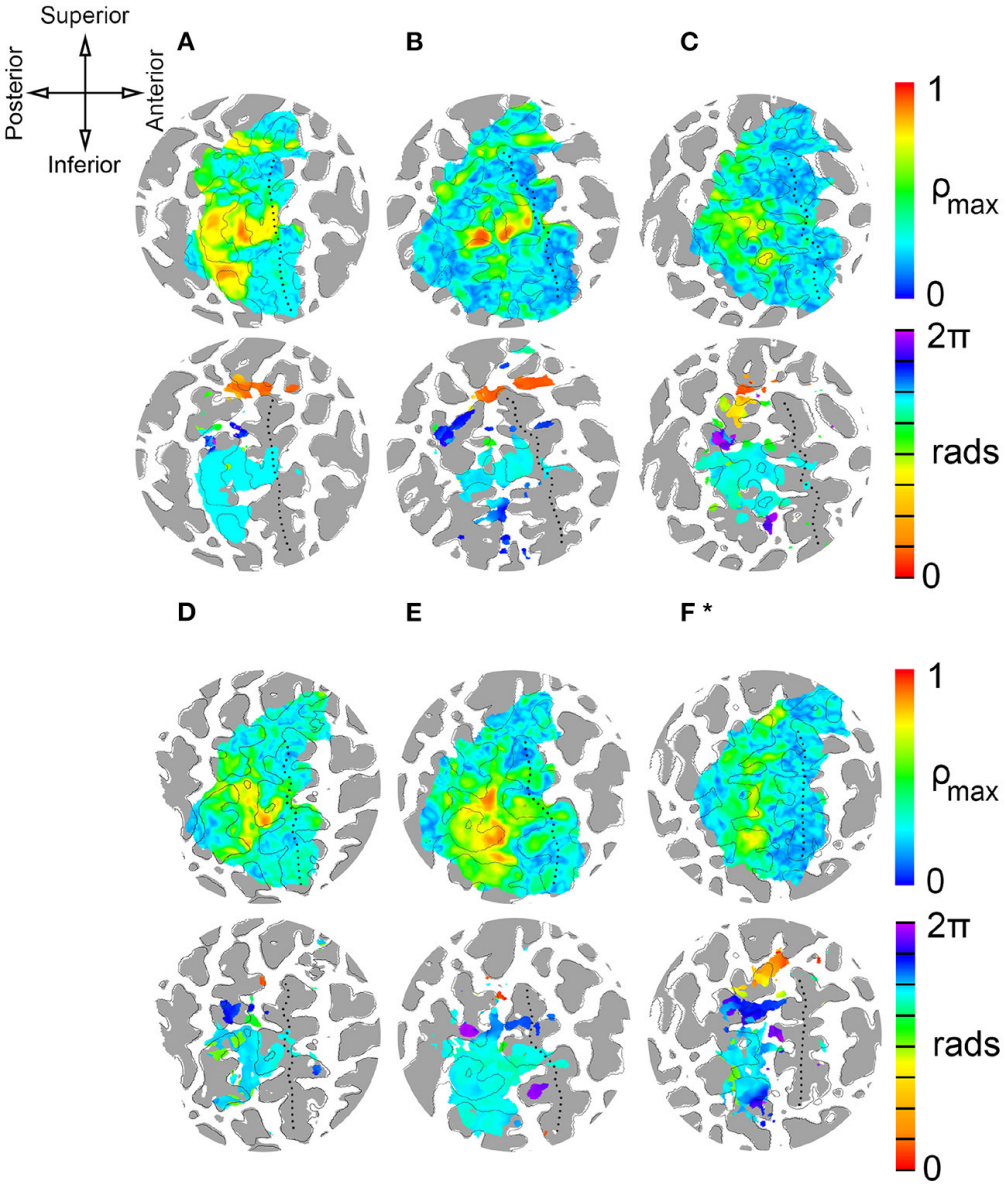
Single-subject results from delay analysis. (A–F)*: Maximum cross-correlation coefficients ρ (top circles) and phase delays Δϕ of cortical surface elements (gray areas are regions of negative convexity). Phase delays are shown for surface elements with average ρ ≥ 0.45. ROI contains central sulcus, postcentral gyrus, postcentral sulcus, and paracentral lobule. (*) Stimulation period for subject F was 40 s (8 × 5 s). Stimulation period for subjects A-E was 72 s (8 × 9 s). Top left: Anatomical orientation of flattened maps. Superior and inferior also correspond to medial and lateral, respectively.

Representative mean time courses from a forward stimulation sequence with participant A are plotted in Figure 3. The top time series is the ideal time course *s*_1_(*t*) with *t*_0_ = 0 s. Each colored line beneath the black ideal series is an average of surface elements within the ROI shown in the flattened maps of Figure 4. The color of each line corresponds to the delay bin those surface elements were assigned to based on which ideal series was maximally correlated to the BOLD response in that surface element. Each time course has the mean value subtracted and is vertically offset for clarity. No additional scaling was applied to the experimental data. The ideal time series was scaled to roughly the same scale as the experimental time courses. The colorbar at the top of Figure 3 depicts the forward stimulation sequence used in the functional run during which these data were acquired.

### 3.2. Individual Somatosensory Maps

Figure 4 presents the results of delay analyses for six participants, labeled A–F, and Table 1 lists the MNI coordinates and mean cross-correlation coefficient of activated clusters. Flattened cortical surface maps of the right hemisphere show regions of negative convexity as dark gray areas. The dotted black lines on the surfaces highlight the central sulcus. The anatomical orientation of the maps is shown on the compass rose in the top-left of the figure. Two maps are presented for each participant: a heat map of maximal cross-correlation coefficients ρ is shown in the top circle, and a phase map with ρ ≥ 0.45 is shown in the bottom circle. Color bars illustrating the scaling of each quantity are on the right side of the figure, and the time delay color scale is partitioned into eight segments, one for each skin area that is illustrated and color-coded on the silhouettes to the left of the figure. The delay analysis was restricted to an ROI based on prior anatomical knowledge of the human primary somatosensory cortex (Penfield and Boldrey, 1937; Roux et al., 2018), encompassing the postcentral gyrus, central sulcus, postcentral sulcus, and paracentral lobule contralateral to peripheral stimulation. The boundaries of the ROI can be seen in the extent of the correlation maps in the top circles.

**Table 1:** Centers of mass of activated clusters.

**Subject A**						**Subject B**					
	**x**	**y**	**z**	**A**	**〈ρ〉**		**x**	**y**	**z**	**A**	**〈ρ〉**
Foot	−6	43	74	200	0.659	Foot	−5	38	70	161	0.551
Shin	−11	47	79	63.8	0.65	Shin	−12	43	80	13.9	0.593
Thigh	–	–	–	–	–		−3	33	67	2.24	0.485
Trunk	–	–	–	–	–	Thigh	−13	47	79	3.18	0.458
Forearm	−17	43	62	26.5	0.572	Trunk	−16	44	74	3.64	0.463
	−35	42	62	10.4	0.622	Forearm	−31	42	72	20.5	0.497
Finger	−47	29	51	1010	0.72		−22	52	68	5.08	0.49
	−19	51	60	20.8	0.616		−10	30	56	3.95	0.517
Neck	−29	39	74	40.1	0.589	Finger	−41	34	62	494	0.638
	−22	49	55	39.7	0.605		−10	29	56	22.9	0.53
Forehead	−22	50	56	17.8	0.599		−26	51	58	2.23	0.469
	−27	40	74	15.2	0.566	Neck	−61	18	41	132	0.497
							−17	39	67	131	0.538
							−4	40	59	8.64	0.483
						Forehead	−18	49	60	15	0.522
**Subject C**						**Subject D**					
	**x**	**y**	**z**	**A**	**〈ρ〉**		**x**	**y**	**z**	**A**	**〈ρ〉**
Foot	−11	48	79	33.3	0.545	Foot	−15	41	78	7.79	0.507
Shin	−12	49	78	2.38	0.467	Shin	–	–	–	–	–
Thigh	−15	44	69	30.2	0.481	Thigh	–	–	–	–	–
Trunk	−15	47	67	33.8	0.504	Trunk	–	–	–	–	–
Forearm	−29	36	50	22.5	0.568	Forearm	−37	38	55	166	0.583
	−32	42	67	17.6	0.494		−11	25	58	3.64	0.499
	−40	25	34	4.68	0.462	Finger	−47	30	58	664	0.626
Finger	−44	32	53	766	0.566		−11	26	57	10.5	0.572
Neck	−21	49	56	15.5	0.523		−21	39	71	7.95	0.491
Forehead	−22	49	55	73.9	0.565	Neck	−30	40	60	238	0.522
	−60	13	34	10.3	0.474		−42	17	48	16.6	0.507
						Forehead	−19	41	60	10.1	0.495
**Subject E**						**Subject F**					
	**x**	**y**	**z**	**A**	**〈ρ〉**		**x**	**y**	**z**	**A**	**〈ρ〉**
Foot	−15	36	79	9.74	0.517	Foot	−11	45	77	39	0.555
	−11	48	78	2.84	0.485	Shin	−14	47	78	80.4	0.608
Shin	–	–	–	–	–	Thigh	−15	45	70	16.1	0.482
Thigh	–	–	–	–	–	Trunk	−17	37	73	13.6	0.483
Trunk	–	–	–	–	–	Forearm	−51	20	24	5.31	0.473
Forearm	−38	36	65	16.9	0.504		−45	21	34	4.42	0.487
	−16	36	78	0.45	0.477	Finger	−50	25	43	335	0.567
Finger	−46	28	48	1340	0.636	Neck	−39	35	54	293	0.534
	−19	36	75	17.3	0.494	Forehead	−26	42	55	59	0.506
Neck	−30	38	70	195	0.534		−54	10	30	2.73	0.456
	−57	9	32	7.89	0.498						
Forehead	−49	20	40	40.5	0.511						
	−22	41	58	34.7	0.536						

*MNI coordinates (x, y, z) [mm], surface area A [mm^2^], and mean cross-correlation coefficient < ρ >. Cluster activation threshold was ρ ≥ 0.45 (α ≥ 0.05). Maximum graph distance between activated surface element and cluster was 10*.

Peak activations, quantified by cross-correlation between the measured BOLD time series and the ideal hemodynamic response for a given stimulus, were apparent about half-way down the postcentral gyrus and postcentral sulcus for each participant near what is considered to be BA 1 and BA 2. Smaller areas of peak activation were observed on the anterior bank of the postcentral gyrus, near BA 3. From the phase maps, it is apparent that these strong activations were due to fingertip stimulation. Maximum cross-correlation coefficients were 0.78 ± 0.08 (95% CI). Lower limb stimulation, including the foot and leg, provoked responses in more superior cortical regions in the paracentral lobule. Stimulation of the more distal foot area was correlated to more anterior regions colored red in Figure 4, while more proximal stimulation was correlated to more posterior cortical regions colored orange. This seems to indicate a proximal-distal posterior-anterior somatotopy of the lower limbs that is apparent in the red-to-orange gradient near the top of each phase map. Trunk stimulation just beneath the left rib-cage correlated to responses in the superior postcentral gyrus and/or sulcus. Stimulation of the left anterior forearm was well correlated to responses near the middle of the anterior bank of the postcentral gyrus and posterior bank of the central sulcus near BA 3, as well as to more superior regions of the postcentral gyrus, near BA 2. Similarly, head and neck stimulation was correlated to two different regions: one superior to the arm and finger representation in the superior one-third of the postcentral region near BA 2 and one inferior to the upper limb representation near BA 3. There is some individual variation in which head and neck representations are most apparent in the results. Participants A and D showed little or no inferior representation of the face and neck, and participant B had very few surface elements with face activation. Somatotopic gradients between neck and head stimulation is apparent in both regions of maps from participants C, E, and F, where forehead-related activation is generally more posterior to neck-related activation.

**Figure 5:**
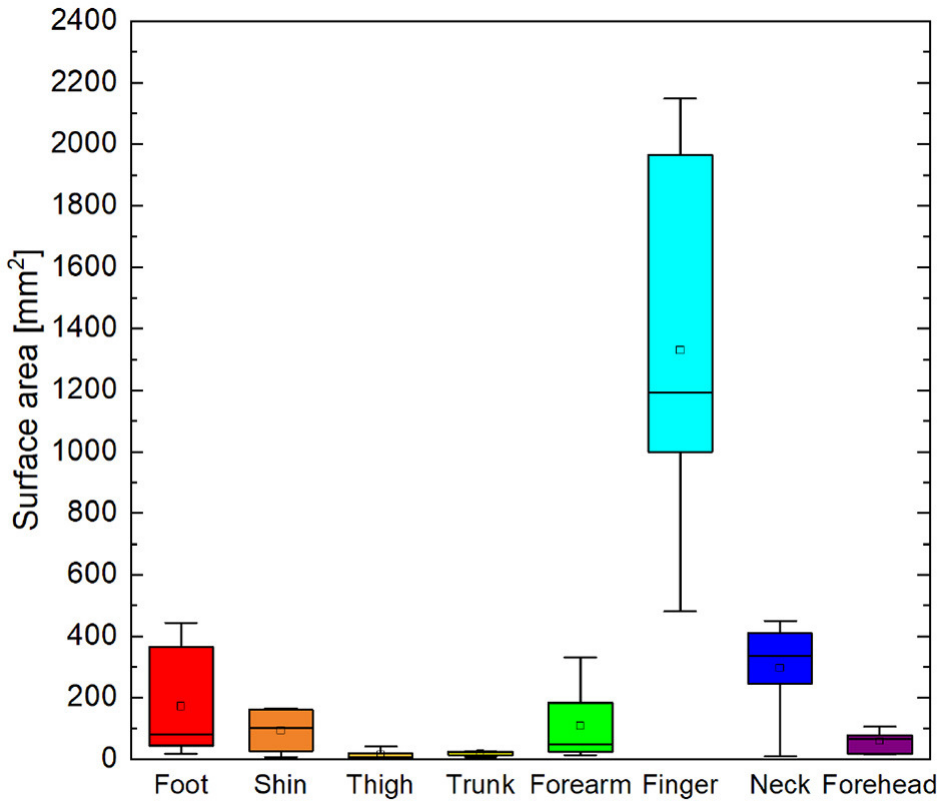
Box and whisker plot of total area of surface elements within ROI correlated to cutaneous stimuli with ρ ≥ 0.45. Boxes delineate 95% confidence interval for each cutaneous area. Median is shown as horizontal line in each box. Whiskers show minima and maxima.

Cortical magnification factors of each body area representation can be surmised from the phase maps in Figure 4 and are reported as cluster surface area in Table 1. Given that each stimulator was positioned such that the wheels traveled over skin to their maximum extent, it can be assumed that the area of skin stimulated at each site was approximately the same, making a comparison between total surface area of cortical activation equivalent to a comparison of cortical magnification factor. Cortical magnification factors for each skin area were then quantified by calculating the total area of surface nodes inside the ROI that correlated with somatic stimulation at ρ ≥ 0.45. The activated cortical surface areas responding to skin stimulation for all participants are plotted in Figure 5. For all subjects, more than one-third of all activated surface elements in the ROI were maximally correlated to fingertip stimulation. The cortical surface area for this skin region was found to be (12 ± 5) cm^2^. For subjects A, B, C, and F, more than half of activated surface elements were due to fingertip stimulation.

### 3.3. Group Somatosensory Map

**Figure 6:**
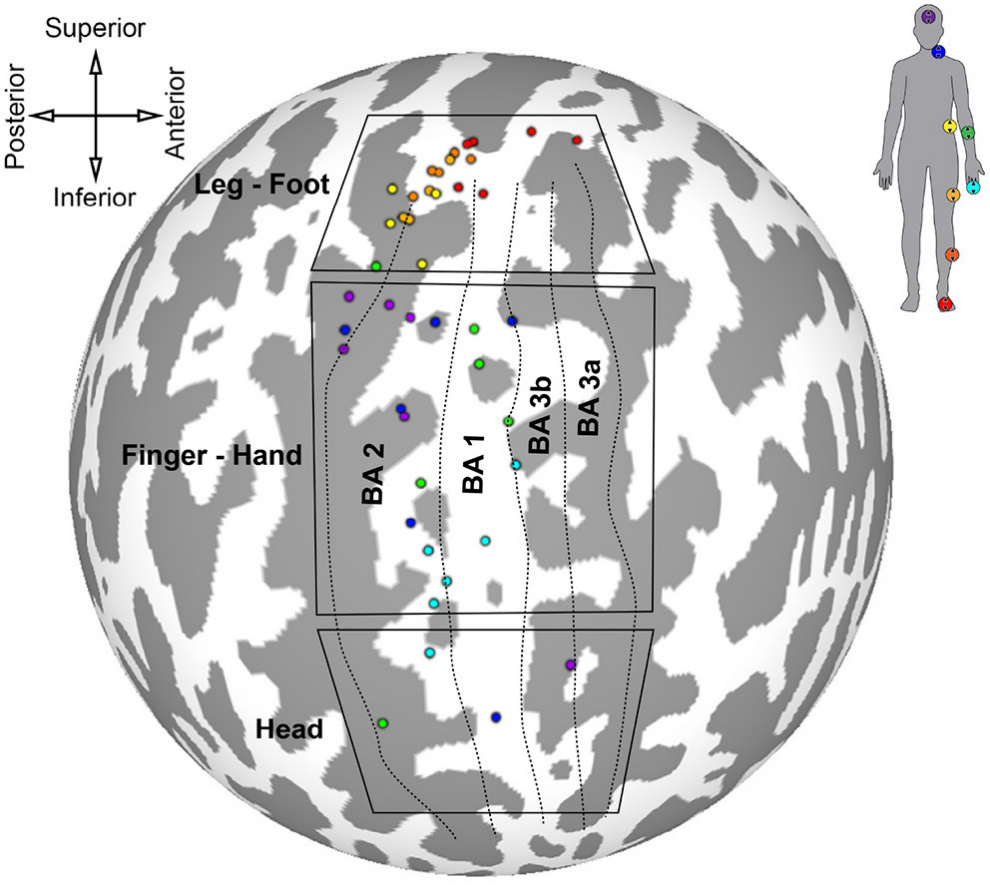
Locations of largest clusters from individual analyses maximally correlated to each peripheral stimuli. Cluster locations are shown on Colin 27 cortical surface map. Colored dots are centered on cluster center of gravity. Labeled boxes delineate regions of interest for certain body areas based on results from Penfield and Boldrey (1937) and Roux et al. (2018). Approximate parcellations of Brodmann's areas are shown as dotted lines. Top-left: anatomical orientation of spherical map. Top-right: color scheme corresponding to stimulator location.

The results of the first somatotopic mapping experiment are summarized on the spherical cortical surface of the Colin 27 template in Figure 6. Centers of mass for the largest maximally correlated clusters were calculated for each participant and for each stimulated skin area, and each center of mass is colored according to which ideal stimulation time series was most correlated to that cluster. Locations of each stimulator and the corresponding color are shown on the silhouette in the top-right of the figure. Anatomical orientation of the flattened map is given in the top-left of the figure. Approximate boundaries of Brodmann's areas 3a, 3b, 1, and 2 are represented by dotted lines, with the most anterior boundary of BA 3a taken to be the fundus of the central sulcus. The most posterior boundary of BA 2 was assumed to be the fundus of the postcentral sulcus. Boxes outline three general regions of interest for lower limb, hand, and head representations derived from two intrasurgical microstimulation studies (Penfield and Boldrey, 1937; Roux et al., 2018).

The cluster centers for each skin area illustrated in Figure 6 were scattered throughout the somatosensory cortex contralateral to stimulation, indicating variation in position of maximally correlated clusters between participants. Activated clusters were found in BA 2, 1, 3b, and 3a. Clusters associated with lower limb stimulation were grouped in the superomedial cortex, or paracentral lobule, wrapping around the longitudinal crest of the hemisphere. Trunk stimulation showed greatest correlation in the postero-superior somatosensory cortex near the top of the postcentral sulcus. Centers of lower limb and trunk representations showed the least amount of intersubject variation. Forearm and finger stimulation were correlated to areas near the center of S1, and forearm-related clusters were the most scattered among participants. Clusters related to head and neck stimulation were mostly localized in the postero-superior area of S1 along the anterior bank of the postcentral sulcus, except for two clusters located in the head ROI, inferior to the finger-hand area.

## 4. Discussion

### 4.1. Large Variability of Individual Somatosensory Maps

For all of the subjects scanned, the BOLD response in a specific region of the brain correlated with tactile stimulation of a skin area, which is consistent with the theory of functional localization as well as previous somatosensory cortex mapping studies. Specific locations associated to stimulation of various skin areas differed on an individual basis, but the overall organization of each somatotopic map was consistent. Lower limb stimulation elicited BOLD responses that were the most consistent among participants. Peak responses to foot and leg stimulation were near the midsagittal plane for most of the participants, within 1 or 2 cm of the longitudinal fissure. Lower limb representations on the phase maps do not cover as much area as other representations, so a greater degree of consistency between individuals could be expected. Trunk, distal arm, head, and neck stimulation generated localized responses that were more scattered among individuals. The total area of activated regions also varied between subjects. The variation between individual maps are likely due to differences in experimental execution from day to day, differences in the awareness of each individual, which was not controlled for in this experiment, or actual somatotopic variations. Future work will need to address intrasubject variability by performing repeated trials of mapping experiments while controlling for intentional awareness.

Most of the activated clusters shown in Figure 6 were located near BA 2, and only three clusters were located in BA 3. Cortical thickness generally decreases in the posterior to anterior direction, from BA 2 to BA 3 (Sanchez-Panchuelo et al., 2014), therefore it is not surprising that more of the largest clusters are located in BA 2, with relatively few in BA 3.

### 4.2. Departures From Penfield's Homunculus

Some features of the somatosensory maps are consistent with the cortical sensory homunculus theory. Cutaneous areas of the lower limb were represented in the superomedial region of S1 near BA 2, BA 1 and BA 3b, consistent with intracranial and neuroimaging experiments (Penfield and Boldrey, 1937; Akselrod et al., 2017; Saadon-Grosman et al., 2020). The forearm and finger were represented further down S1, near the level of the motor hand knob (Schweisfurth et al., 2018). Face and neck representations were found in inferior regions of S1, however these representations were only found in four of the subjects studied and they were generally not dominant clusters, i.e., they had relatively low cross-correlation coefficients compared to other face and neck-related activations. S1 regions with overall highest levels of correlation to face and neck stimulation were found in the superior one-third of the postcentral sulcus, superior to representations of the fingertip and inferior to those of the lower limb. Most of the clusters that showed activation due to head and neck stimulation were in the finger-hand ROI. This is a major departure from the summary maps of Penfield et al., but is consistent with the facial representation on S1 in macaques using microelectrode recording and with more recent findings of an additional face representation using fMRI on humans (Penfield and Boldrey, 1937; Dreyer et al., 1975; Sanchez-Panchuelo et al., 2018). This is the first fMRI-based evidence of multiple head-neck representations in the human S1.

The additional head-neck representation begs the question of whether somatotopic data from intracranial stimulation is comparable to data acquired from neuroimaging modalities that measure cortical activity in relation to peripheral somatic input. With the former method, no information is relayed to or from the peripheral nervous system, while with the latter method, information is passed in the usual manner from periphery to central nervous system, including all of the decussations and processing waypoints that may exist between them. Any conclusions drawn from the two methodologies may differ, which would highlight future avenues of inquiry related to sensory processing, sensation, and perception. Another important difference in the two approaches to somatotopic mapping is that direct cortical stimulation of awake patients can also only be done on the pial surface, generally near the apex of a gyrus. Deep sulcal tissue would not be readily accessible to electrodes, so it is possible that these areas in the postcentral sulcus may elicit a subjective perception if they were able to be stimulated.

The scatter in maximally correlated clusters shown in Figure 6 suggests that group-level statistics at the voxel or surface element level are unsatisfactory for a comprehensive understanding of somatosensory maps. Individual somatotopies do not necessarily map onto one another, even when moving to a standard space. A more detailed analysis of group-level statistics and the relation to individual maps will need to be carried out.

### 4.3. Large Variability of Somatosensory Cortical Magnification

Cortical magnification of different cutaneous areas was highly variable from person to person. The fingertip elicited robust, widespread hemodynamic responses in the somatosensory cortices of all participants. As seen in Figure 5 the finger representation was on the order of 10 cm^2^, nearly 25% of the total surface area of the postcentral gyrus and sulcus. For participant F, the surface area was about 15% less, while for participant D, the surface area was only 3 cm^2^. The cortical surface area of other representations also varied widely between individuals. Again, the variation may be due to actual somatotopic differences between people or confounding factors such as variations in skin sensitivity, awareness, stimulator positioning, and bunching or pinching of straps that may have changed the effective area of stimulation.

### 4.4. Limitations of This Study and Future Work

While care was taken to ensure each stimulator was attached at the same relative point on each participant's body and was attached in the same way, it is possible that stimulator intensity for a particular skin area differed between subjects leading to a source of intersubject variability in percent change of BOLD signal due to tactile stimulation. Also, during scanning, participants may have shifted and caused a change in relative stimulation intensity leading to a source of intrasubject variability. Additional experiments carried out in the same way on the same participant would have provided valuable information about this variability.

A deterministic sequence, either forward or reverse, was used for each of the functional imaging runs. No pseudorandom ordering was used, which may have affected the robustness of stimulation for later runs. Participant expectations and desensitization to stimuli may have had a confounding influence on the results, which may limit rigorous comparison of data collected in the same imaging session. Several studies, both fMRI and PET based, have investigated the effects of expectations on the processing of sensory input. One study by Drevets et al. suggested that anticipation of a localized tactile stimulus decreases cerebral blood flow in areas of cortex not responsible for processing input from the area in which stimulation is anticipated (Drevets et al., 1995). This implies that utilizing a predictable sequence for stimulation would result in decreased BOLD contrast due to reduction in blood flow throughout the somatosensory system.

A useful extension to this work includes measuring individuals' somatosensory thresholds and the use of stimuli above and below these pressures. Additional studies may examine the directional nature of afferent neurons by using linear actuators at different orientations relative to the skin surface. Other sensory modalities may also be mapped and compared to maps generated using light touch stimuli, including hot, cold, and different vibratory frequencies.

## 5. Conclusion

fMRI was used to non-invasively quantify the neurovascular response of cortical tissue in S1 for six participants. An MR-safe, automated pneumatic device was developed to carry out tactile stimulation at several different sites. Activation was estimated as the maximum cross-correlation coefficient at a certain phase shift between ideal time series and measured BOLD time courses. Centers of gravity for maximally correlated clusters were calculated, and cortical magnification factors were estimated from measurements of cortical surface area that correlated to peripheral stimulation with correlation coefficients >0.45.

Resulting maps shared some features with the accepted theory of the sensory homunculus, including superomedial lower limb representations in S1 and upper limb and finger representations at the level of the motor hand knob. However, one important distinction was the forehead and neck representation being along the top one-third of the postcentral sulcus, superior to upper limb representations. This finding contradicts Penfield's “upside-down” homunculus and is the first fMRI evidence of multiple head-neck representations in S1 in multiple human subjects.

## Data Availability Statement

The raw data supporting the conclusions of this article will be made available by the authors upon reasonable request.

## Ethics Statement

The studies involving human participants were reviewed and approved by The University of Alabama at Birmingham Institutional Review Board. The patients/participants provided their written informed consent to participate in this study.

## Author Contributions

WW and MB designed the experiments. WW and KT collected the data. WW analyzed the data and drafted the manuscript. WW, KT, and MB interpreted the analysis, edited, and revised the manuscript. All authors contributed to the article and approved the submitted version.

## Conflict of Interest

The authors declare that the research was conducted in the absence of any commercial or financial relationships that could be construed as a potential conflict of interest.
